# Noninvasive, Transient and Selective Blood-Brain Barrier Opening in Non-Human Primates In Vivo

**DOI:** 10.1371/journal.pone.0022598

**Published:** 2011-07-22

**Authors:** Fabrice Marquet, Yao-Sheng Tung, Tobias Teichert, Vincent P. Ferrera, Elisa E. Konofagou

**Affiliations:** 1 Department of Biomedical Engineering, Columbia University, New York, New York, United States of America; 2 Department of Neuroscience, Columbia University, New York, New York, United States of America; 3 Department of Psychiatry, Columbia University, New York, New York, United States of America; 4 Department of Radiology, Columbia University, New York, New York, United States of America; National Institute of Health, United States of America

## Abstract

The blood-brain barrier (BBB) is a specialized vascular system that impedes entry of all large and the vast majority of small molecules including the most potent central nervous system (CNS) disease therapeutic agents from entering from the lumen into the brain parenchyma. Microbubble-enhanced, focused ultrasound (ME-FUS) has been previously shown to disrupt noninvasively, selectively, and transiently the BBB in small animals *in vivo*. For the first time, the feasibility of transcranial ME-FUS BBB opening in non-human primates is demonstrated with subsequent BBB recovery. Sonications were combined with two different types of microbubbles (customized 4–5 µm and Definity®). 3T MRI was used to confirm the BBB disruption and to assess brain damage.

## Introduction

The main limiting factor towards the development of novel treatments of neurological and neurodegenerative diseases is the blood-brain barrier (BBB): more than 98% of small-molecule drugs and nearly all large-molecule drugs do not cross this anatomic barrier [1], [2]. Several techniques exist to circumvent the BBB, such as intracranial injections, mixing or attaching agents to BBB-modifying chemicals, and the chemical alteration of agents to be delivered through endogenous transport systems [2], [3]. However, these techniques are either invasive, drug-specific or are plagued by very poor spatial specificity. Even the latest advances in brain gene therapy [4] provide cell specific drug delivery but are not region specific. Global breaching of the BBB can be a risky process, as it increases influx of all molecules and therapeutic agents in untargeted areas of the brain [5] even if this approach has been proven to be successful for some applications such as metastatic lung cancer [6]. An ideal method would ensure drug-independent, reversible, localized and noninvasive delivery through the BBB to minimize potential hazards. Previously, our group has shown, along with others, that microbubble-enhanced, focused ultrasound (ME-FUS) is capable of disrupting the BBB noninvasively, transiently and selectively in small animals [7], [8], [9] typically at frequencies above 1 MHz. Although the exact mechanism of BBB opening remains to be determined, the interaction of the ultrasound beam with microbubbles results in mechanical perturbation of the microvasculature and thereby changes in the integrity of the BBB components. Previous studies suggest that both paracellular and transcellular barriers are affected during and after ME-FUS exposure [10].

Until now, however, the method has mainly been confined to research laboratories without specific prospects for clinical translation [1] because feasibility in large mammals has not been shown. Demonstration in large animals can open new avenues in targeted delivery of BBB-impermeable therapeutic agents (*e.g.*, nerve growth factor, gene therapy), which have been shown effective in the treatment of neurodegenerative diseases such as Alzheimer's and Parkinson's [11], [12], [13] and have been shown feasible in small animals [14], especially since early detection has been shown lately to be feasible using PET imaging [15], warranting focal and noninvasive treatment methodologies. This technique could also prove useful for basic neuroscience research, replacing invasive techniques such as intracranial microinjections.

The BBB is a complex regulatory system within the neurovascular unit, which controls the flow of nutrients and chemicals into and out of the brain parenchyma maintaining the brain homeostasis necessary for proper neuronal firing [16]. The BBB hinders the effective systemic delivery of neurological agents and biomarkers to the brain through a combination of passive, transport and metabolic barriers. Determining factors for the passage of molecules across the BBB are lipid solubility, charge and molecular size (threshold range spans between 50 Da and 400 Da) [17]. Therefore, potential therapeutic agents, such as inhibitors to enzymes (100–1,000 Da) and proteins (30–3,000 kDa), do not efficiently cross the BBB when administered systemically. Such delivery and efficacy are critical in inducing therapeutic effects and triggering biological pathways.

Applying ME-FUS BBB opening in large animals is very challenging as focusing inside the brain is impeded by the presence of the skull along the beam path. The big difference between the high speed of sound through the skull and the low speed through the underlying brain tissue, combined with a severe attenuation of ultrasound waves through the skull bone, strongly distorts the beam shape especially at higher frequencies [18]. High intensity focused ultrasound (HIFU), a promising technique used in noninvasive tumor ablation, has led the way of transcranial focusing since the early 1950s [19]. The interest of HIFU on CNS diseases treatment was initiated in the 1950s [20], but for both of these applications total craniotomy was performed to circumvent the skull attenuation and aberration effects. The introduction in the 1990s of piezoceramic and piezocomposite transducers capable of being driven at high voltages and the improvement of multichannel electronics [19] have allowed to overcome these effects and provide accurate focusing through the skull in the late 1990s [21], [22], [23]. Transcranial HIFU research has recently paved the way for the development of complex, high cost and efficient methods requiring prior knowledge of the skull topology to perform accurate focusing [24], [25] with transcranial feasibility shown for ablation shown in humans [26], [27], [28]. While HIFU therapy use continuous wave (CW) and relies on thermal effects in order to induce a thermal necrosis, ME-FUS BBB opening uses short pulsed-wave (PW) and relies mostly on mechanical effects such as cavitation (be it stable or inertial) [29], [30].

An alternative to correcting for the aberrations induced by the skull is to operate at lower frequencies, but the focus can become very wide due to diffraction effects, thus decreasing the spatial resolution. Sonothrombolysis studies use transcranial CW ultrasound to dissolve clots in the brain, at typically lower frequencies (around 200 kHz), which are less prone to phase aberrations and absorption but may enhance cavitational effects [31], [32], [33]. The beam is generally loosely focused to cover a large volume of the brain in each application. However, one of these studies showed large, secondary hemorrhage [34], which has been hypothesized to be linked to unexpected enhanced cavitation effects caused by standing waves generated within the skull [35], [36]. Standing waves are known to be capable of trapping microbubbles in the antinodes and decreasing their inertial cavitation threshold [36], [37]. ME-FUS BBB opening also relies on mechanical effects to transiently and locally increase the trans-BBB permeability but uses PW sequences with very short duty cycles (from 0.1% to 2%). Therefore, the safety should be easier to ensure despite the use of low frequencies (around 200 kHz).

Our group has thus selected a middle solution to the aforementioned tradeoff, *i.e.*, operate at intermediate frequencies (500 kHz) that allows transcranial propagation and sufficiently high spatial resolution with a single-element transducer, warranting a sufficiently wide safety window. Until now, feasibility with this system has been shown in simulations and *in vitro* [38], [39].

## Results and Discussion

In this study, transcranial ME-FUS is shown for the first time to induce BBB disruption in non-human primates (NHP). A total of four locations were disrupted in two animals (see Methods). Two neighboring regions in the visual cortex (V3), the caudate and the hippocampus were targeted. Pressures ranging from 0.3 MPa to 0.6 MPa were investigated. Previous studies have shown that a pressure increase results in a larger BBB opening extent and higher BBB permeability [41] while a safety window exists within the pressure range of 0.30 MPa (threshold of opening) and 0.60 MPa [42]. For all experiments, T1-weighted MRI at 3.0T was used to confirm the BBB disruption, tracking the diffusion of intravenous (IV) injected gadodiamide in the brain.

**Figure 1:**
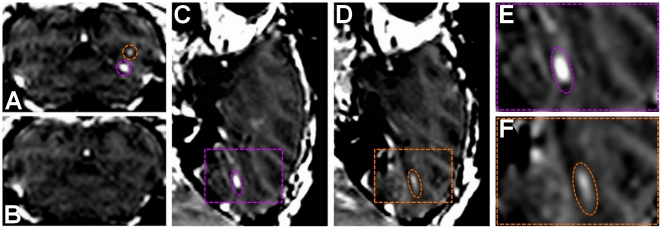
BBB opening in V3. A 3D Spoiled Gradient-Echo (SPGR) T1-weighted sequence was applied after intravenous (IV) injection of gadodiamide 1 h after sonication. Gadodiamide presence in the brain parenchyma is induced by BBB opening. (**A**) Coronal MR images confirming the local disruption of the BBB, two opening sites are circled confirmed by the diffusion of the contrast agent (purple circle 0.3 MPa and orange circle 0.45 MPa). (**B**) Coronal MR images 6 days, after sonication, confirming closing of the BBB and proving reversibility of the procedure. (**C**,**D**) Sagittal MR images of the two sonication sites at two different pressures 0.3 MPa (**C**) and 0.45 MPa (**D**). (**E**,**F**) Magnification of the corresponding color boxes.

**Figure 2:**
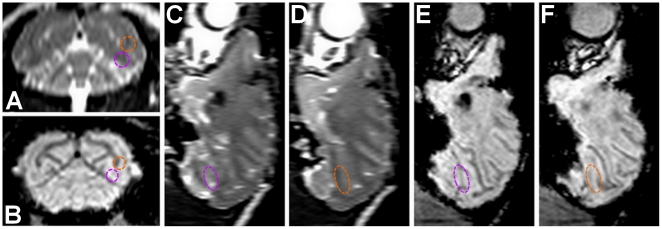
Damage assessment. (**A**,**C**,**D**) 3D T2-weighted sequence. Edemas should appear brighter in these images. (**B**,**E**,**F**) 3D Susceptibility-Weighted Image (SWI) sequence was applied. Hemorrhages, as well as large vessels should appear in black in these images. (**A**,**B**) Same reconstructed coronal slice as shown in figure 1. The two opening sites are circled with the corresponding colors. There is no difference between the two hemispheres. (**C**,**D**,**E**,**F**) Corresponding reconstructed sagittal slices for the two opening sites. No oedemas or hemorrhages are visible.

Figure 1 depicts the results obtained for the visual cortex targets. The spatial selectivity of ME-FUS was hereby investigated by inducing BBB disruption in two neighboring, distinct, small sites in the visual cortex region at two different ultrasonic pressures (0.3 MPa and 0.45 MPa). The contrast agent cannot penetrate the BBB, therefore the deposition of the gadodiamide in the parenchyma confirmed local BBB disruption by ME-FUS (Fig. 1A,C,D). The MR images indicated that the BBB was opened at both 0.3 MPa (Fig. 1A bottom site and Fig. 1C) and 0.45 MPa (Fig. 1A top site and Fig. 1D). The peak MR intensity enhancement at the BBB-opened region relative to the average value in the parenchyma was increased by 119% and 48% at 0.3 MPa and 0.45 MPa, respectively. The volume of the BBB disruption was equal to 24.6 mm^3^ and 30.5 mm^3^, respectively. The two distinct opened sites were separated by 4.74 mm. A higher density of microbubbles at the ME-FUS focus for the 0.3 MPa site may have been caused by the proximity to a larger vessel, explaining the higher MRI contrast enhancement. The location of the induced BBB disruption areas were shifted from the expected location of respectively 0.8 mm and 0.7 mm laterally and 8.1 mm and 7.9 mm axially towards the transducer. The same MRI sequence and IV contrast agent injection were repeated six days after BBB opening (Fig. 1B). No intensity enhancement was observed indicating that the BBB was closed or reinstated. Two other MRI sequences (T2-weighted and susceptibility-weighted) were used to assess potential brain damage after ME-FUS and both of them indicated absence of detectable damage such as edema or hemorrhage (Fig. 2).

**Figure 3:**
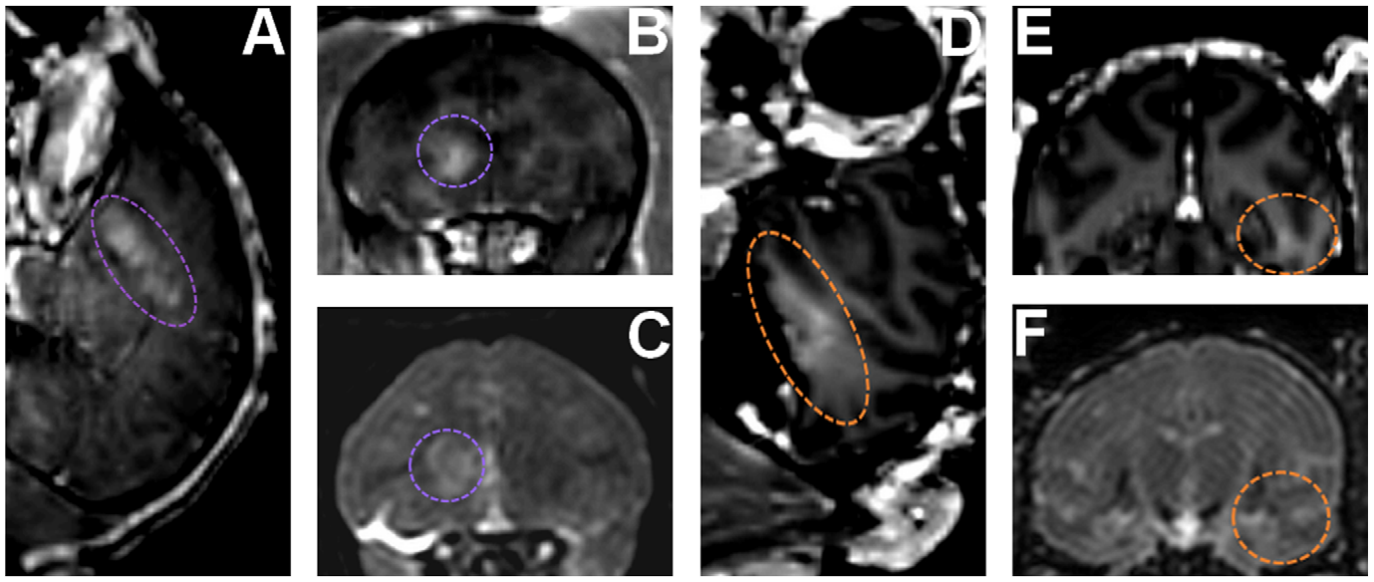
BBB opening in caudate and hippocampus & damage assessment. (**A**,**B**,**C**) BBB opening experiment targeting caudate using customized microbubbles and applying 0.6 MPa (purple dashed line shows region of interest). (**D**,**E**,**F**) BBB opening experiment targeting hippocampus using Definity® microbubbles and applying 0.6 MPa (orange dashed line shows region of interest). (**A**,**B**,**D**,**E**) 3D Spoiled Gradient-Echo (SPGR) T1-weighted sequence was applied after intravenous (IV) injection of gadodiamide 1 h after sonication. (**A**,**D**) Sagittal slices at the region of interest. (**B**,**E**) Corresponding coronal slices. (**C**,**F**) 3D T2-weighted sequence, an edema was visible using customized microbubbles while no damage was detected using Definity® microbubbles.

The same protocol was repeated for the two following sessions applying 0.6 MPa and two different kinds of microbubbles. The results are shown in Fig. 3. T1-weighted MR sequences were used to track the diffusion of gadodiamide. Using both of these microbubbles we obtained larger BBB disruption areas (Fig. 3A,B,D,E). This is mainly because by increasing the peak pressure, a larger portion of the brain reaches the disruption threshold. The peak MR intensity enhancement at the BBB-opened region relative to the average value in the parenchyma was increased by 68% and 41% using customized and Definity® microbubbles, respectively. The volume of the BBB disruption was equal to 285.5 mm^3^ and 116.3 mm^3^, respectively. The BBB opening regions at the caudate and the hippocampus were shifted from the targeted location by respectively 0.6 mm and 0.9 mm laterally and 6.5 mm and 7.2 mm axially. T2-weighted MR sequences were also used to assess potential damages in the brain (Fig. 3C,F). An edematous region was detected using custom made microbubbles while no damage was detected using Definity®. All the animals have been survived and therefore histological findings are not available at this time. Even though no in-depth cognitive tests have been performed thus far, qualitative assessment of the animal basic behavior has been monitored. Normal cognitive behavior has been noted following ME-FUS procedures at moderate pressures and using Definity®. In the case of 0.6 MPa and customized microbubbles, the animal with the edema exhibited a weakness in the contra-lateral arm over four days after treatment, most likely due to the induced edema, but then fully recovered after that four-day period.

**Figure 4:**
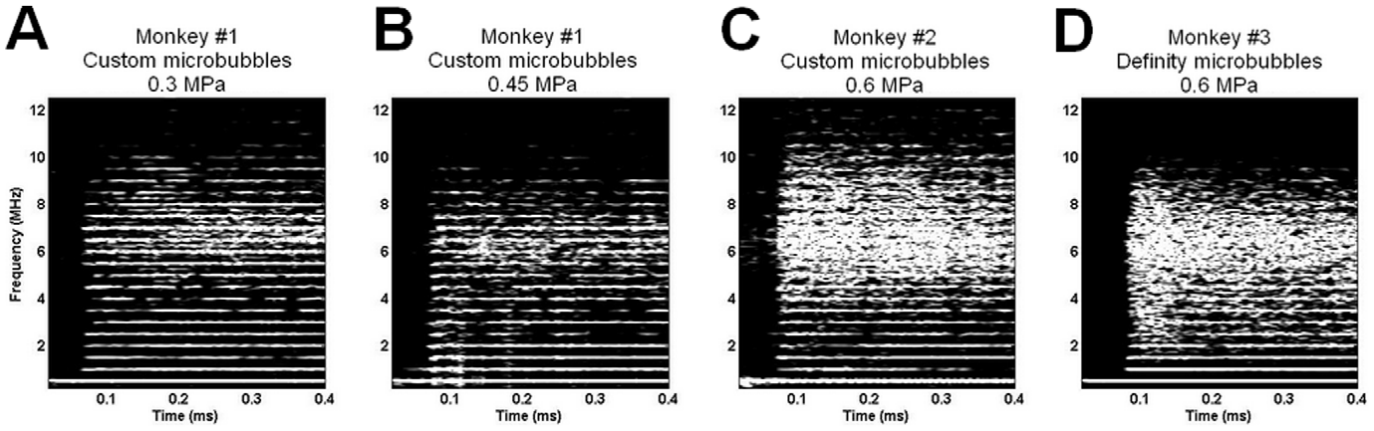
PCD recordings during ME-FUS exposure. The corresponding spectrograms of the first pulse with microbubbles administration shows that very few broadband acoustic emissions are detected at 0.3 MPa (**A**) and 0.45 MPa (**B**) using customized microbubbles. This suggests that in those cases, the mechanism is mainly dominated by stable cavitation. Increasing the pressure to 0.6 MPa, using both customized (**C**) and Definity® (**D**) microbubbles, a large amplitude broadband signal (4–8 MHz) is detected which is the signature of inertial cavitation.

Passive cavitation detector (PCD) recordings were performed during all experiments and are depicted in Fig. 4. Spectrograms depicted the frequency content of the bubble response during ME-FUS application and helped classify the cavitation behavior. Using moderate pressures (Fig. 4A,B), the PCD recordings showed the nonlinear modes due to the bubble oscillations induced by the acoustic excitation (stable cavitation). Very little broadband response was detected. This noise is induced by bubble collapse and jet, more generally described as inertial cavitation. In those two cases, the cavitation behavior was mainly dominated by stable cavitation. While increasing the pressure to 0.6 MPa, a large broadband signal was recorded for both customized (Fig. 4C) and Definity® (Fig. 4D) microbubbles. Previous work from our group has shown that inertial cavitation is one of the main causes of induced damage during treatment [41]. We believe that the discrepancy observed in the last two experiments is mainly due to the bubble size dependence. A recent study [43] showed that contrary to current belief, liquid jets are directed away from the nearest vessel wall. Therefore, using smaller microbubbles, those jets might not actually be able to puncture and damage the vessels while using larger microbubbles trapped between vessel walls, jets are more likely to induce damage. Customized and Definity® microbubbles do not only differ in size. The gas is also different (perfluorobutane for customized and perfluoropropane for Definity®) but since the solubility and diffusivity of those two gases are similar, it is not expected to significantly affect the bubble oscillation during ME-FUS exposure. The different carbon chains (DSPC and DPPC, respectively), however, may change the shell property. The effect of shell property is part of ongoing work in our lab in order to assess the role of microbubbles in BBB opening.

Achieving drug-independent, localized, reversible and noninvasive BBB disruption in non-human primates can pave the way towards novel brain drug delivery and gene therapy techniques. The volume of the BBB opening was shown to be small enough using moderate pressures (24.6 mm^3^ and 30.5 mm^3^) to ensure potential therapeutic agents in untargeted regions. This technique was also proven to be highly selective with two distinct BBB opening sites in the visual cortex separated by a distance on the order of a few millimeters. Increasing the pressure resulted in larger opening regions (285.5 mm^3^ and 116.3 mm^3^). Those preliminary results have also enlightened the brain site and microbubbles type dependences. The targeting quality was also assessed. These results were found to be in good agreement with previous *in silico* and *in vitro* findings [38], [39]. These previous studies showed that as long as we choose a good targeting vector (with an incidence angle close to normal), the distortion and lateral shift are very low, the attenuation and axial shift are also reproducible *in vitro* (with less than 1 dB and 1 mm of standard deviation respectively) at the same angle of incidence. The initial *in vivo* results suggest that the shift induced by the skull interface may also be reproducible *in vivo* under the same angle of incidence condition. Other targeting vectors might require a more complex way of determining the position of the focus and the global attenuation (*e.g.*, simulations based on prior 3D CT skull scans). Ongoing work will be achieved in order to assess those dependences in a statistically significant way. At this point, no quantification of the contrast agent diffusion was performed. In future work, a permeability quantification technique reported in mice by our group [44] will be applied as part of the primate study.

In conclusion, initial feasibility of noninvasive, highly selective, drug-independent and reversible BBB opening was demonstrated in non-human primates *in vivo* for the first time. High spatial selectivity of this technique was also shown. This study is a major step toward clinical translation of this emerging technology that can be combined with any type of pharmacological treatment to the brain. Ongoing investigations entail optimization of the procedure including safety and efficacy of the trans-BBB drug delivery.

## Materials and Methods

Initial feasibility studies were performed on two male rhesus macaques over the course of three sessions. In the first two sessions, the monodispersed 4–5 µm microbubbles were manufactured in-house and size-isolated using differential centrifugation [40] while in the last session Definity® microbubbles were used (Lantheus Medical Imaging, MA, polydispersed, mean diameter 1.1–3.3 µm, 98% below 10 µm, maximum diameter 20 µm). In the first session, a region of the visual cortex (ventral V3), *i.e.*, the medio-ventral wall of the occipital cortex, was targeted. In the second animal (second session), the caudate was targeted and the pressure was increased to 0.6 MPa to investigate potential damage induced using customized microbubbles. The third and final session was performed in the first animal targeted the hippocampus using 0.6 MPa and Definity® microbubbles.

**Figure 5:**
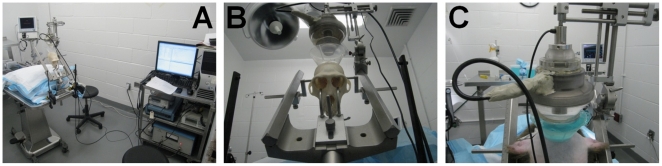
Experimental setup for *in vivo* FUS-induced BBB opening in the operating room. (**A**) A single-element, circular focused ultrasound transducer with a hole in the center was driven by a function generator (Agilent Technologies, Palo Alto, CA, USA) through a 50-dB power amplifier (ENI Inc., Rochester, NY, USA). The center frequency, focal depth, outer radius and inner radius of FUS were 500 kHz, 90 mm, 30 mm and 11.2 mm, respectively. (**B**) Closer view of the transducer mounted on the stereotactic frame with a manipulator allowing precise positioning of the transducer in the stereotactic referential. (**C**) Monkey placed in the stereotactic frame. The monkey head is shaved and a degassed echographic gel container is placed on the top of its head to insure maximal acoustic transmission.

A 500-kHz center frequency focused ultrasound transducer was used for this experiment (Riverside Research Institute, NY, USA). The acoustic parameters used for the three protocols were the following: focal maximum estimated pressure of 0.3 MPa and 0.45 MPa, pulse length of 10 ms, pulse repetition frequency of 2 Hz, total sonication duration of 2 min. The single-element transducer was mounted on a standard monkey stereotactic frame for accurate positioning (Fig. 5). *In vitro* pressure measurements were realized in another study [39]. This study determined the global attenuation (absorption, reflexion and scattering) due to the presence of the skull (around -5.7 dB at 500 kHz). The attenuation in the skin [45] was assumed to be around −0.9 dB.cm^−1^ and its thickness was estimated to be equal to 0.5 cm. The attenuation in the monkey brain tissue [45] was assumed to be around −0.5 dB.cm^−1^ and the thickness of this layer was estimated to be equal to 2 cm. Therefore, the emission amplitude has to be raised by 7.15 dB (approximately a factor 2.28) compared to the calibration measurements in water to compensate for the energy loss along the path.

The first animal had previously participated in several electrophysiological and fMRI experiments. It had a surgically implanted head post to restrain head movements. The head post was embedded within a dental-acrylic implant which was held in place by ceramic screws. The screws penetrated the skull plate but did not protrude more than a millimeter into the skull-cavity. A previously implanted scleral search coil had been removed prior to the experiments reported here. During the electrophysiological experiments, single unit activity was recorded from the frontal eye fields of both hemispheres. No recording from the animal's brain targeted regions had been previously performed. The dental-acrylic implant covered a large portion of the skull. However, the occipital pole of the skull was not covered and enabled a sufficient acoustic window to the visual cortex without interference from the implant.

**Figure 6:**
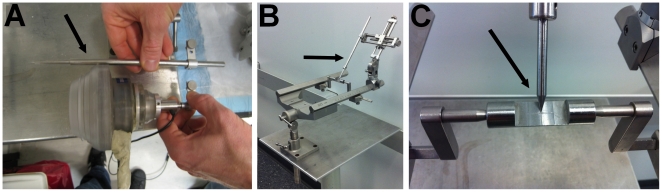
Targeting procedure for *in vivo* FUS-induced BBB opening. (**A**) A positioning rod (black arrow), indicating the position of the focus (5 cm away from the edge of the transducer), was used to target. (**B**) This positioning rod was mounted on the manipulator in order to locate the origin of the stereotactic coordinates. (**C**) The origin of the stereotactic coordinates indicated by the engraved cross on the metal piece between the ear-bars is targeted with the tip of the positioning rod.

For the application of the FUS, all animals were anesthetized with 2% isoflurane (carrier gas: oxygen). The heart rate was held at approximately 120 beats per minute and the respiratory rate at around 60 breaths per minute. Prior to sonication, the scalp hair was removed with a depilatory cream to ensure maximal acoustic transmission. The animal's head was then placed in a stereotactic frame to enable careful targeting of the ultrasound. The sonication was performed immediately after intravenous (IV) injection of 500 µL microbubbles for all experiments (5×10^9^ numbers/mL for customized microbubbles and 1.2×10^10^ numbers/mL for Definity®). Targeting was ensured using a manipulator and a positioning rod indicating the position of the focus relatively to the stereotactic coordinates (Fig. 6). Targeted regions of visual cortex (V3) were determined using a monkey brain atlas.

MRI was used to confirm BBB opening using gadiodiamide contrast agent. 3D Spoiled Gradient-Echo (SPGR) T1-weighted sequences (TR/TE = 20/1.4 ms; flip angle: 30°; NEX = 2; spatial resolution: 500×500 µm^2^; slice thickness: 1 mm with no interslice gap) were applied after intravenous (IV) injection of gadodiamide (Omniscan®, molecular weight 573.66 Da, GE Healthcare, Princeton, NJ, USA) 1 h after sonication. The dose applied was 0.2 mL/kg and the IV injection was performed 2 minutes before the SPGR T1-weigthed scan (scan duration: 18 minutes). Gadodiamide presence in the brain parenchyma was induced by BBB opening. 3D T2-weighted sequence (TR/TE = 3000/80; flip angle: 90°; NEX = 3; spatial resolution: 400×400 µm^2^; slice thickness: 2 mm with no interslice gap) and 3D Susceptibility-Weighted Image (SWI) sequence were applied (TR/TE = 19/27 ms; flip angle: 15°; NEX = 1; spatial resolution: 400×400 µm^2^; slice thickness: 1 mm with no interslice gap) and were used to assess brain damage.

A single-element PCD (center frequency: 7.5 MHz, focal length: 60 mm, Olympus NDT, Waltham, MA, USA) was positioned through the center hole of the FUS transducer. The two transducers were aligned so that their focal regions fully overlapped within the confocal volume. The PCD transducer, which was connected to a digitizer (Gage Applied Technologies, Inc., Lachine, QC, Canada) through a 20-dB amplification (5800, Olympus NDT, Waltham, MA, USA), was used to passively acquire acoustic emissions from microbubbles. A time-frequency map of the acoustic emission was generated using a customized spectrogram function (8-cycles, *i.e.*, 16 µs, Chebyshev window; 98% overlap; 4096-point FFT) in MATLAB® (2010a, Mathworks, Natick, MA). The spectrogram can then clearly indicate how the frequency content of the signal changes over time. Therefore, the presence of broadband response can help classify cavitation behavior.

### Ethical statement

All animal studies were approved by the Institutional Animal Care and Use Committee at Columbia University and the New York State Psychatric Institute (protocol ID: CU1066, NYSPI279). All animals, housed and handled in strict accordance with good animal practice under supervision of veterinarians, received environmental enrichment and were monitored for evidence of disease and changes in attitude, appetite, or behavior suggestive of illness. Every effort was made to alleviate animal discomfort and pain by appropriate and routine use of anesthetic and/or analgesic agents.
